# The MicroRNA-23b/27b/24 Cluster Facilitates Colon Cancer Cell Migration by Targeting FOXP2

**DOI:** 10.3390/cancers12010174

**Published:** 2020-01-10

**Authors:** Kensei Nishida, Yuki Kuwano, Kazuhito Rokutan

**Affiliations:** Department of Pathophysiology, Institute of Biomedical Sciences, Tokushima University Graduate School, 3-18-15 Kuramoto-cho, Tokushima 7708503, Japan; kuwanoy@tokushima-u.ac.jp (Y.K.); rokutan@tokushima-u.ac.jp (K.R.)

**Keywords:** cell migration, *miR-23b/27b/24* cluster, *C9orf3*, FOXP2

## Abstract

Acquisition of cell migration capacity is an early and essential process in cancer development. The aim of this study was to identify microRNA gene expression networks that induced high migration capacity. Using colon cancer HCT116 cells subcloned by transwell-based migrated cell selection, microRNA array analysis was performed to examine the microRNA expression profile. Promoter activity and microRNA targets were assessed with luciferase reporters. Cell migration capacity was assessed by either the transwell or scratch assay. In isolated subpopulations with high migration capacity, the expression levels of the *miR-23b/27b/24* cluster increased in accordance with the increased expression of the short *C9orf3* transcript, a host gene of the *miR-23b/27b/24* cluster. E2F1-binding sequences were involved in the basic transcription activity of the short *C9orf3* expression, and E2F1-small-interfering (si)RNA treatment reduced the expression of both the *C9orf3* and *miR-23b/27b/24* clusters. Overexpression experiments showed that *miR-23b* and *miR-27b* promoted cell migration, but the opposite effect was observed with *miR-24*. Forkhead box P2 (FOXP2) mRNA and protein levels were reduced by both/either *miR-23b* and *miR-27b*. Furthermore, FOXP2 siRNA treatment significantly promoted cell migration. Our findings demonstrated a novel role of the *miR-23b/27b/24* cluster in cell migration through targeting FOXP2, with potential implications for the development of microRNA-based therapy targeted at inhibiting cancer migration.

## 1. Introduction

Cell migration plays a pivotal role in epithelial–mesenchymal transition (EMT), invasion, or metastasis in various types of tumors. Tumors can adapt to microenvironments, resulting in heterogeneous subpopulations with distinct properties of proliferation, migration, invasion, or sensitivity to therapies. As a consequence of cellular adaptation, some tumor cells lose their epithelial characteristics, gain mesenchymal properties, and aggressively migrate to the non-tumorigenic extracellular matrix, and are described as EMT. Cell migration is an essential property of EMT and can be a therapeutic target for inhibiting metastatic progression [1].

MicroRNAs (miRNAs) are small non-coding RNAs (containing 21–22 nucleotides), which promote either mRNA degradation or block their translation through binding to partially complementary sequences of their target mRNAs. A single miRNA has more than 100 target mRNAs, and post-transcriptionally regulates their expression profiles. Therefore, altered expression patterns of even a small number of miRNAs could influence cell phenotype, including tumorigenesis and malignant transformation, through regulation of a wide range of biological processes [2,3]. A miRNA cluster is a set of more than two miRNAs produced from a single primary transcript. miRNAs belonging to clusters coordinately regulate multiple processes through targeting functionally related proteins [4,5,6,7]. The *miR-23b/27b/24* cluster is composed of three miRNA genes located within an intron of *C9orf3* gene on human chromosome 9q22.32. Although previous studies have investigated the roles of *miR-23b/27b/24* miRNAs in tumor progression, conflicting functions of *miR-23b/27b/24* miRNAs have been reported during tumor development and metastatic progression [8,9]. Inhibition of *miR-23b* has been shown to decrease proliferation, migration, and invasion in nasopharyngeal carcinoma by directly targeting E-cadherin [10]. Down-regulation of *miR-27b* inhibits cell growth and invasion in cervical cancer cells [11]. Both *miR-23b* and *miR-27b* cooperatively regulate Nischarin expression, resulting in the promotion of tumorigenic properties in breast cancer cells [12]. Conversely, several studies reported that either *miR-23b* or *miR-27b* acts as a tumor suppressor in breast and colorectal cancers [8,9]. Most research on *miR-23b/27b/24* miRNAs has focused on the roles of individual miRNAs in regulating specific target genes. However, potential coordinated effects of the *miR-23b/27b/24* cluster on tumor progression are not fully understood. Furthermore, based on the knowledge of intronic miRNAs biogenesis, the pri-miR-23b/27b/24 cluster could be transcribed as part of the transcript of the host gene, *C9orf3*. The precise mechanism of regulation of the *miR-23b/27b/24* cluster expression has not been investigated.

In this study, using a subpopulation with high migration capacity isolated from HCT116 cells using transwell apparatus [13], we sought to identify the *miR-23b/27b/24* cluster, whose expression was upregulated in a subpopulation with cell migration capacity. The promoter assay of *C9orf3*, the host gene of the *miR-23b/27b/24* cluster, revealed that E2F1 was involved in the regulation of the basic transcription activity of the short *C9orf3* transcript. Furthermore, we identified forkhead box P2 (FOXP2) as a novel target for both *miR-23b* and *miR-27b*. The *miR-23b/27b/24* cluster may promote, at least in part, cell migration by regulating FOXP2 expression.

## 2. Results

### 2.1. Identification of miRNAs Responsible for the High Migration Capacity

**Figure 1:**
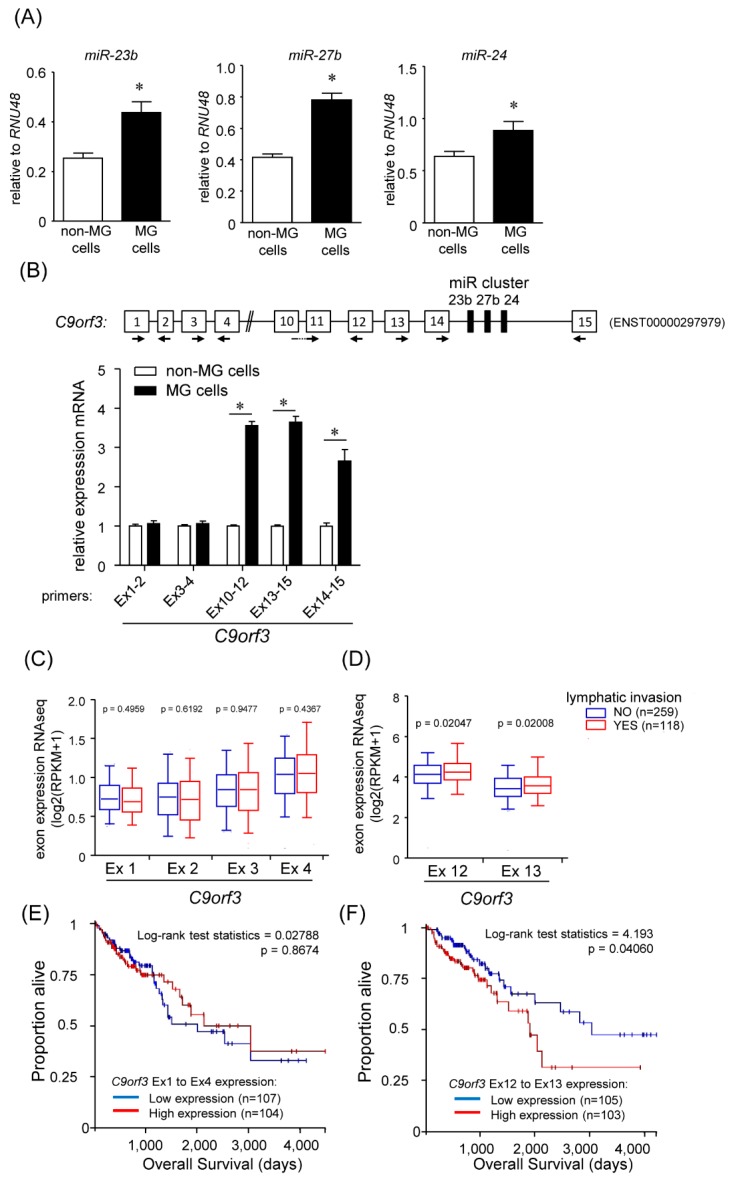
Up-regulation of the *miR-23b/27b/24* cluster expression in migrated (MG) cells. (**A**) Relative expression levels of *miR-23b*, *miR-27b*, and *miR-24* in non-MG cells and MG cells were measured by RT-qPCR. *RNU48* was used as an endogenous control. (**B**) mRNA levels of *C9orf3*, a host gene of *miR-23b/27b/24* cluster, were measured by real-time reverse transcription polymerase chain reaction (RT-qPCR) using the indicated primer sets. Data are expressed as the mean fold changes ± standard deviation (SD; n = 4), compared with those in the non-MG cells. * Statistically significant difference versus non-MG cells (unpaired Student’s *t*-test, *p* < 0.05). (**C**,**D**) Samples from TCGA (Colorectal Adenocarcinoma, COADREAD) were divided into two groups according to the presence or absence of lymphatic invasion. The difference in gene expression of each exon in *C9orf3* between the subgroups was tested for significance using Welch’s *t*-test on log-transformed data. Individual mRNA levels are presented on box-and-whiskers plots using a logarithmic scale for the *y*-axis. (**E**,**F**) Overall survival as a function of *C9orf3* expression in TCGA. Patients with *C9orf3* expression data from TCGA (COADREAD) were evenly divided into quartiles, and the lowest and highest quartiles were plotted with Kaplan-Meier curves for overall survival using the UCSC Xena browser tool.

We have previously succeeded in isolating a subpopulation with accelerated baseline motility (migrated cells [MG] cells) and an immotile one (non-MG cells) from a colon cancer cell line (HCT116 p53 wild type) [13]. The MG cell subpopulation was composed of EMT intermediates with high expression levels of EMT marker genes *ZEB1* and *VIM* [13]. In addition, MG cells expressed surface markers of colorectal cancer stem cells (*ALDH1A1*, *CD24*, *POU5F1*, *SOX2*, and *SOX9*) to a greater degree compared with non-MG cells [14] (Figure S1). Using these subpopulation cells, we investigated novel regulatory miRNAs involved in cell migration. To identify miRNAs differentially expressed in the MG cells, we prepared total RNA samples from the MG and the non-MG cells, respectively. These samples were subjected to miRNA microarray analysis, and miRNA expression profiles were compared between MG and non-MG cells. Of the 939 probes, 124 probes were passed over the threshold of the “Flag at Present.” Followed by further filtering of the raw signal intensity of 50 in at least one sample, 56 human miRNAs remained. Using the mean expression change of 1.5-fold criterion, we identified four miRNAs (*miR-10a*, *miR-23b*, *miR-27b*, and *miR-1274b*) differentially expressed between MG and non-MG cells (Table 1). Because *miR-1274b* is defined as a dead entry on the miRBase (Release 21), *miR-1274b* was excluded from further analysis. We validated the miRNA expression of *miR-10a*, *miR-23b*, and *miR-27b*. *miR-23b* and *miR-27b* belong to the same miR-cluster, which contains *miR-23b*, *miR-27b*, and *miR-24*. Therefore, we also measured *miR-24* expression levels besides *miR-23b* and *miR-27b*. The amounts of *miR-23b*, *miR-27b*, and *miR-24* in MG cells were significantly higher than those in non-MG cells (Figure 1A). However, we could not detect the sufficient expression of *miR-10a* in both the MG and non-MG cells.

### 2.2. Transcription of Short C9orf3 Isoforms in the MG Cells

The *miR-23b/27b/24* cluster is located at intron 14 of *C9orf3* transcript (ENST00000297979). Because the expression levels of all three members of the *miR-23b/27b/24* cluster were upregulated in MG cells, we investigated changes in the gene expression of a host gene of the *miR-23b/27b/24* cluster, *C9orf3*. Using five primer sets designed for amplification of different segments of the *C9orf3* transcript, we measured *C9orf3* expression levels by real-time reverse transcription polymerase chain reaction (RT-qPCR). Although both MG and non-MG cells expressed similar amounts of amplified products containing exon 1 to 2 or exon 3 to 4 of *C9orf3* mRNA, amounts of amplified products containing exon 10 to 12, exon 13 to 15, or exon 14 to 15 of *C9orf3* mRNA were significantly increased about 3-fold in the MG cells (Figure 1B). Furthermore, we evaluated the clinical relevance of gene expression differences within the *C9orf3* gene. As shown in Figure 1C,D, although the expression levels of *C9orf3* exons 1 to 4 were similar irrespective of the presence or absence of lymphatic invasion in colorectal adenocarcinoma, those of *C9orf3* exons 12 and 13 were significantly upregulated in samples with lymphatic invasion. Furthermore, patients with high expression levels of *C9orf3* exons 12 and 13 exhibited a lower overall survival than patients with lower expression levels. In contrast, the expression levels of *C9orf3* exons 1 to 4 did not affect overall survival (Figure 1E,F). These data suggest that the mRNA levels of the latter part of *C9orf3* are associated with cell migration or invasion and lead to poor prognosis. The discrepancy in exon expression levels in *C9orf3* is consistent with the presence of short *C9orf3* transcripts (containing exons 10–15) in MG cells. To confirm this hypothesis, we employed 5′-rapid amplification of cDNA end (RACE) analysis using primers of *C9orf3* exon 14 as detailed in Figure S2. Eighteen clones were isolated and sequenced by the Sanger method, and we identified two novel transcriptional start sites (TSSs), TSS1, and TSS2. Of 18 clones, 13 clones corresponded to TSS2 sequence, suggesting that TSS2 was preferentially used as the first exon in MG cells (Figure S2A,B). Indeed, TSS2 expression levels were three times higher than those of TSS1, detected by absolute quantification of gene expression using RT-qPCR (Figure S2C). Therefore, we focused on TSS2-containing short *C9orf3* transcript in further analyses.

### 2.3. Regulation of Short C9orf3 Expression

#### 2.3.1. Effect of DNA Methylation on C9orf3 and *miR-23b/27b/24* Expression

Hovestadt et al. performed a comprehensive assessment of the correlation between DNA methylation and gene expression; they predicted the presence of a short *C9orf3* transcript in WNT-pathway activated medulloblastoma [15]. They also showed that the expression of either *C9orf3* or individual miRNAs of the *miR-23b/27b/24* cluster is negatively correlated with CpG island methylation. To evaluate the effect of DNA methylation on the expression of individual miRNAs of the *miR-23b/27b/24* cluster, we measured these expression levels after 48 h treatment with 5-azacytidine (5-aza), a DNA methyltransferase inhibitor. The levels of each microRNA of the miR-*23b/27b/24* cluster were rather decreased in the non-MG cells. Additionally, the mRNA expression of TSS1 and TSS2 in short *C9orf3* was not affected by 5-aza treatment. These data suggested that DNA methylation was neither involved in the transcriptional regulation of short *C9orf3* mRNA nor *miR-23b/27b/24* miRNA expression in our isolated HCT116 cell lines (Figure S3).

#### 2.3.2. Promoter Array of Short *C9orf3* Transcript

**Figure 2:**
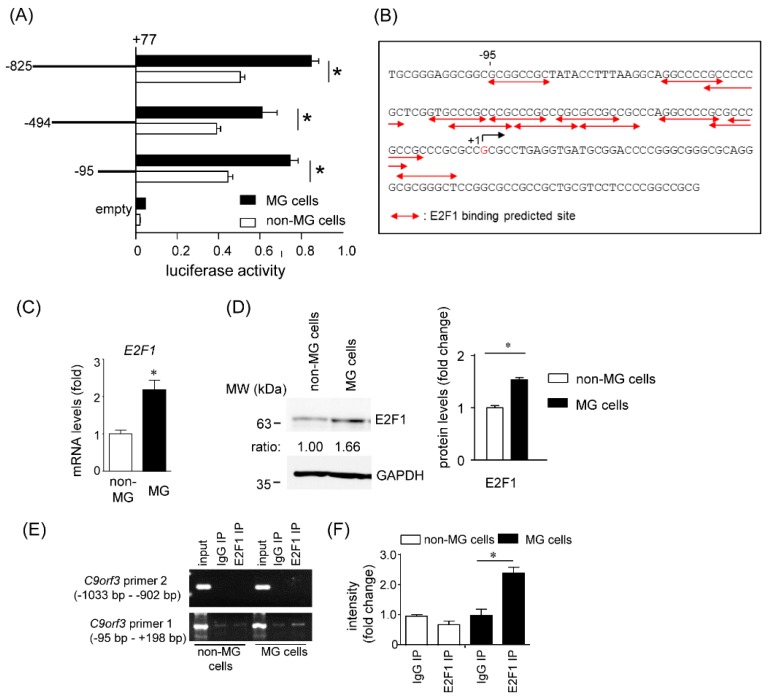
E2F1 regulates *C9orf3* promoter activity. (**A**) HCT116 cells were transiently transfected with luciferase reporter plasmids driven by the indicated promoter fragments of *C9orf3* for 48 h. Luciferase activities in these cells were measured using the Dual-Luciferase Reporter Assay System. * Significant up-regulation in MG cells compared with non-MG cells. Data are expressed as the mean ± standard deviation (SD; n = 4). * Statistically significant difference versus non-MG cells (unpaired Student’s *t*-test, *p* < 0.05). (**B**) Nucleotide sequence of the 5′-flanking region of the *C9orf3* gene. Putative binding sites for E2F1 are indicated with bent arrows in red based on the PROME. (**C**) *E2F1* mRNA levels were measured by RT-qPCR using the indicated primer sets. Data are expressed as the mean fold changes ± standard deviation (SD; n = 4), compared with those in the non-MG cells. * Statistically significant difference versus non-MG cells (unpaired Student’s *t*-test, *p* < 0.05). (**D**) Amounts of E2F1 proteins in the non-MG cells and MG cells were measured by western blotting using glyceraldehyde-3-phosphate dehydrogenase (GAPDH) as a loading control. The results of western blotting using anti-E2F1 antibody from three independent experiments were quantified by densitometry as shown in (**D**). Data are expressed as the mean ± standard deviation (SD; n = 4). * Statistically significant difference versus non-MG cells (unpaired Student’s *t*-test, *p* < 0.05). (**E**,**F**) HCT116 cells were subjected to chromatin immunoprecipitation (ChIP) assays. Formaldehyde-crosslinked nuclear extracts were immunoprecipitated with an anti-E2F1 antibody or normal rabbit IgG (IgG). PCR was performed using an input nuclear chromatin fraction as a template (input). Specific PCR products corresponding to the region of the *C9orf3* promoter containing E2F1-binding sites were amplified and separated by agarose gel electrophoresis followed by ethidium bromide staining. The results of ChIP with an anti-E2F1 antibody from three independent experiments were quantified by densitometry as shown in (**F**). Data are expressed as the mean ± standard deviation (SD; n = 4). * Statistically significant difference versus control (IgG) (unpaired Student’s *t*-test, *p* < 0.05). MW, molecular weight.

Next, we investigated the mechanism underlying the transcriptional regulation of the TSS2-containing short *C9orf3* transcript. To determine the minimal promoter region of the short *C9orf3* transcript, we generated a luciferase reporter vector having the 5′-flank of the short *C9orf3* from −825 to +77 bp, and then serially truncated the fragments that were constructed. As shown in Figure 2A, the basal promoter activity was found to reside in −95 to +77 bp fragment sequence. Potential transcription factor binding sites within the −95 to +1 bp in the short *C9orf3* promoter were predicted using ALGGEN PROMO version 3.0.2 [16,17]. The region was a GC-rich sequence (86% GC), and a number of E2F1 binding sites (13 sites) were top-ranked (Figure 2B). Increased E2F1 expression has been reported to be associated with cell migration and tumor development [18,19,20]. Indeed, MG cells expressed E2F1 mRNA and protein to a much greater degree compared with non-MG cells (Figure 2C,D). To reveal whether E2F1 interacted with the promoter region in vivo, chromatin immunoprecipitation (ChIP) assay was employed. Cross-linked chromatin from both the MG cells and non-MG cells was immunoprecipitated using anti-E2F1 antibody or normal rabbit IgG. We designed PCR primers to amplify the section of the promoter region of *C9orf3* (−95 to +198 bp), which contained putative E2F1-binding sequences. As a negative control for the ChIP assay of E2F1, we designed a primer set for the *C9orf3* promoter region (−1033 to −902 bp), where E2F1-binding sequence does not exist. The PCR products of E2F1-immunoprecipitated chromatin were amplified with a primer set of the promoter region of *C9orf3* (−95 to +198 bp), only in the MG cells (Figure 2E). Thus, ChIP assay suggested the preferential binding of E2F1 to the short *C9orf3* promoter region in the MG cells.

### 2.4. Effects of E2F1 on C9orf3 and miR-23b/27b/24 Expression

**Figure 3:**
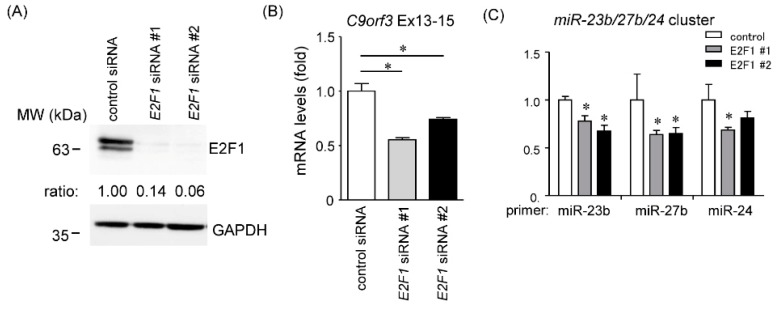
Knockdown of E2F1 decreased *C9orf3* expression levels. (**A**) Two siRNAs targeted for distinct E2F1 sequences were transfected to the MG cells. Amounts of E2F1 proteins in E2F1 siRNAs-treated cells were measured by western blotting using GAPDH as a loading control. (**B**,**C**) Relative expression levels of *C9orf3* and *miR-23b/27b/24* miRNAs in MG cells after treatment with E2F1-siRNAs were measured by RT-qPCR. *GAPDH* or *RNU48* was used as an endogenous control. Data are expressed as the mean ± standard deviation (SD; n = 4). * Statistically significant difference versus control cells (unpaired Student’s *t*-test, *p* < 0.05). MW, molecular weight.

The results of the promoter assay of *C9orf3* and ChIP with anti-E2F1 antibody suggested E2F1 as a potent transcriptional activator of the short *C9orf3* transcript. We measured *C9orf3* mRNA and *miR-23b/27/24* miRNA expression in the MG cells when treated with two small-interfering (si)RNAs, targeting different sites of E2F1 over 48 h. These siRNAs effectively reduced E2F1 protein levels (Figure 3A). Both *C9orf3* mRNA levels and miR-23b/27b/24 miRNAs were significantly decreased in these cells (Figure 3B,C). However, the additional inhibitory effect of E2F1 knockdown on miR-23b/27b/24 expression was not observed in non-MG cells expressing E2F1 at relatively low levels (Figure S4).

### 2.5. Effects of the miR-23b/27b/24 Cluster on Cell Migration

#### 2.5.1. Effect of *miR-23b*, *miR-27b*, and *miR-24* on Cell Migration

**Figure 4:**
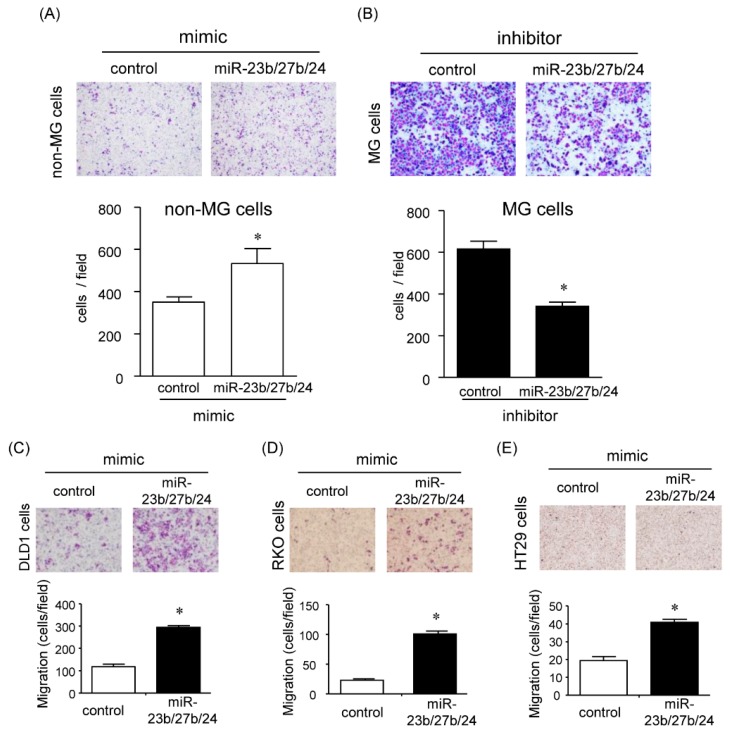
Effects of miR-23b/27b/24 on cell migration. (**A**,**B**) Transwell migration (n = 4) assays were performed in mixed mimic or inhibitor of microRNAs (*miR-23b*, *miR-27b* and *miR-24*)-transfected HCT116 cells. Upper panels show representative images of Diff-Quick staining in four experiments of transwell migration assays, and a lower graph shows quantification of cell migration expressed by cell counting. (**C**–**E**) Transwell migration assays were performed in mixed mimic of microRNAs (*miR-23b*, *miR-27b*, and *miR-24*)-transfected DLD1, RKO, or HT29 cells. Upper panels and lower graphs are the same as in (aA). Values represent mean ± SD. n = 4. * Statistically significant difference versus control (unpaired Student’s *t*-test, *p* < 0.05).

Previous studies showed the pleiotropic functions of each miRNA member of the *miR-23b/27b/24* cluster in cancer. They can function as oncogenes or tumor suppressors depending on the context. To reveal the effects of *miR-23b/27b/24* cluster on cell migration, we assessed the cell migratory capacity using transwell assay. As shown in Figure 4A and Figure S5A overexpression treatment with mixed miRNAs of *miR-23b*, *miR-27b*, and *miR-24* in both non-MG cells and MG cells enhanced the cell migration capability. Consistent with this effect, inhibitory treatment with mixed miRNA inhibitors of *miR-23b*, *miR-27b*, and *miR-24* in MG cells, but not in non-MG cells, significantly reduced the cell migration capability (Figure 4B and Figure S5B). Furthermore, we obtained similar effects of *miR-23b*, *miR-27b*, and *miR-24* during migration in other colon cancer cells; DLD1, RKO, and HT-29 cells (Figure 4C–E).

#### 2.5.2. Effect of a Single MicroRNA on Cell Migration

**Figure 5:**
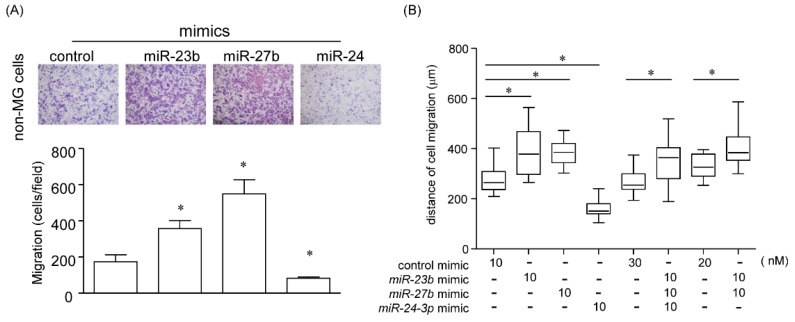
*miR-23b* and *miR-27b* promote cell migration. Transwell assay (**A**) and Scratch assay (**B**) were performed in non-MG cells with combinational transfection of mimic *miR-23b*, *miR-27b*, and/or *miR-24*. (**A**) Upper panels show representative images of Diff-Quick staining in four experiments of transwell migration assays, and a lower graph shows quantification of cell migration expressed by cell counting. Data are expressed as the mean ± standard deviation (SD; n = 4). * Statistically significant difference versus control (unpaired Student’s *t*-test, *p* < 0.05). (**B**) After transfection with mimic miRNAs, cells were scratched. Migration distance was measured at 24 h later. Data are expressed as the mean ± standard deviation (SD; n = 4). * Statistically significant difference versus control (unpaired Student’s *t*-test, *p* < 0.05).

We also confirmed that for the effects of individual miRNAs of *miR-23b/27b/24* cluster on cell migration, transwell (Figure 5A) and scratch assays (Figure 5B) were employed. As shown in Figure 5A, mimics *miR-23b* and *miR-27b* enhanced cell migration, whereas *miR-24* attenuated the cell migratory capability. We employed combinational transfections mimicking *miR-23b*, *miR-27b*, and/or *miR-24*. As shown in Figure 4 and Figure 5B, all three mimicking miRNA (*miR-23b/27b/24*) treatment modalities and the combinational treatment of the mimic *miR-23b* and/or *miR-27b* significantly enhanced cell migration. However, a single treatment of mimic *miR-24* rather inhibited the cell migration; suggesting that *miR-23b* and *miR-27b*, but not *miR-24*, may be involved in cell migration capacity (Figure 5A,B).

### 2.6. Identification of Common Target mRNAs of miR-23b and miR-27b

**Figure 6:**
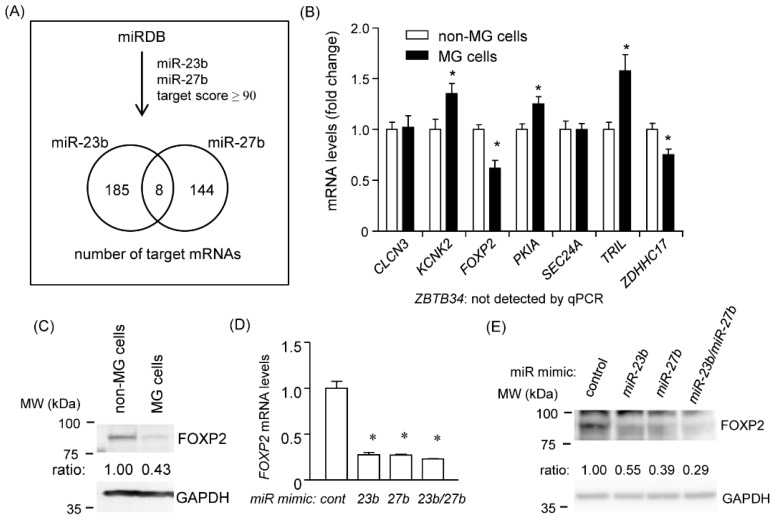
FOXP2 as a potential target of *miR-23b* and *miR-27b*. (**A**) The number of candidate target mRNAs of *miR-23b* or *miR-27b* based on miRDB is shown in a Venn diagram. (**B**) mRNA levels of *CLCN3*, *KCNK2*, *FOXP2*, *PKIA*, *SEC24A*, *TRIL*, *ZDHHC17*, and *ZBTB34* were determined using RT-qPCR in non-MG cells and MG cells. Expression of *ZBTB34* mRNA was not detected by RT-qPCR. (**C**) Amounts of FOXP2 protein in non-MG cells and MG cells were determined using western blotting with GAPDH as a loading control. (**D**) *FOXP2* mRNA levels were determined in non-MG cells with mimic miR-23b and/or miR-27b transfection and RT-qPCR. (**E**) Amounts of FOXP2 protein in non-MG cells with mimic miR-23b and/or miR-27b transfection were determined using western blotting with GAPDH as a loading control. The results of western blotting with an anti-FOXP2 antibody were quantified by densitometry, and the ratio of FOXP2 to GAPDH is shown in (**E**). MW, molecular weight.

Because *miR-23b* and *miR-27b* promoted cell migration capacity, we searched for the possible target mRNAs for these two miRNAs using miRDB. It is known that members of the cluster miRNAs often synergistically regulate the same target mRNA. We therefore focused on mRNAs with putative target sites for both *miR-23b* and *miR-27b*. Based on miRDB, we picked up 193 and 152 genes as *miR-23b* and *miR-27b* targets, respectively. Among these genes, we selected eight candidate genes: *CLCN3*, *KCNK2*, *FOXP2*, *PKIA*, *SEC24A*, *TRIL*, *ZDHHC17*, and *ZBTB34* (Figure 6A). Validation of the expression of these mRNAs in the non-MG and MG cells by qRT-PCR showed that *FOXP2* and *ZDHHC17* mRNA expression was significantly downregulated in the MG cells, suggesting that these mRNAs were possible mRNA targets of *miR-23b* and *miR-27b* (Figure 6B). It has been reported that FOXP2 is associated with the development of several types of cancers [21,22,23]. FOXP2 protein levels were significantly reduced in the MG cells compared with those in the non-MG cells (Figure 6C). The overexpression of mimic *miR-23b* and/or *miR-27b* decreased *FOXP2* mRNA and protein levels (Figure 6D,E), suggesting that *miR-23b* and *miR-27b* may regulate FOXP2 expression.

### 2.7. miR-23b and miR-27b Directly Target the 3′ UTR of FOXP2

**Figure 7:**
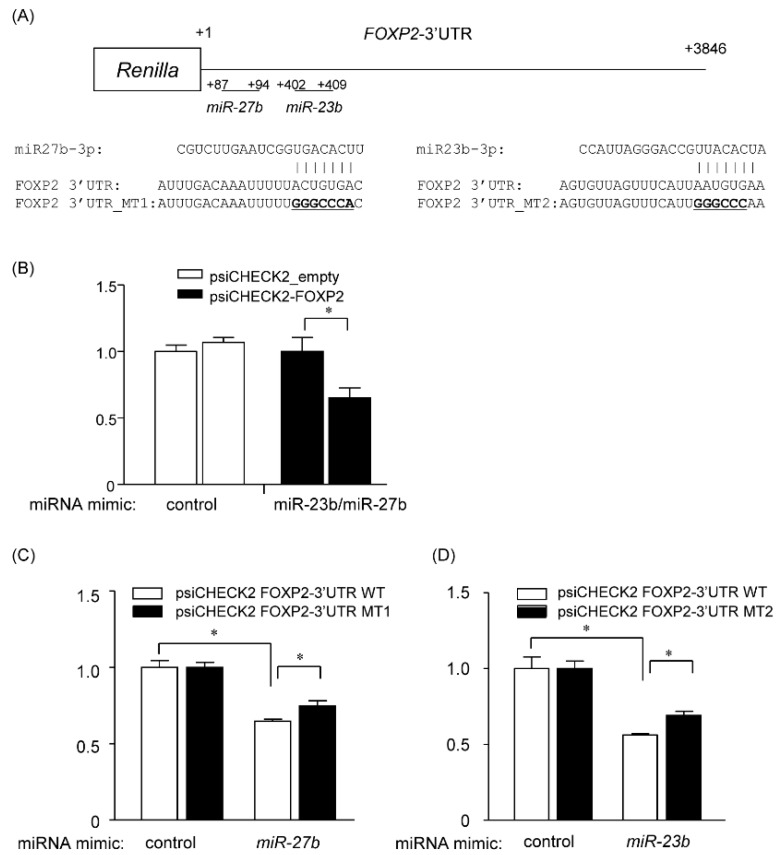
FOXP2 is directly targeted by both *miR-23b* and *miR-27b*. (**A**) miRDB predicted a putative binding site of *miR-23b* (nt 402–409) and of *miR-27b* (nt 87–94) in the *FOXP2* 3’ UTR region. The mutated (MT) sequences of the putative binding sites are shown as *FOXP2* 3′UTR_MT1 or MT2. (**B**) psiCHECK2 vector containing the full length of 3′ UTR in *FOXP2* or empty vector was co-transfected with both *miR-23b* and *miR-27b*. Renilla luciferase activity was normalized to firefly luciferase. Fold-change values were normalized to empty vector. (**C**,**D**) psiCHECK2 vector containing the full length of 3′ UTR in *FOXP2* (psiCHECK2 FOXP2-3′UTR WT) or the mutated binding sequences (psiCHECK2 FOXP2-3′UTR MT1 or MT2) were co-transfected with either mimic *miR-23b* or *miR-27b*. Renilla luciferase activity was normalized to firefly luciferase. Fold-change values were normalized to empty vector. Fold-change values were then normalized to mimic control-treated cells. Data are expressed as the mean ± standard deviation (SD; n = 4). * Statistically significant difference versus control (**B**–**D**) or WT (**C**,**D**) (unpaired Student’s *t*-test, *p* < 0.05).

The miRDB program predicted the putative binding sites for *miR-23b* and *miR-27b* in the 3′ UTR of FOXP2 at nt 402–409 and nt 87–94, respectively (Figure 7A). To verify the potential binding sequences of *miR-23b* and *miR-27b*, we prepared the reporter plasmids (psiCHECK2) that expressed chimeric RNAs containing the sequence for Renilla and 3′ UTR sequence of *FOXP2*, and evaluated the effects of miRNAs on the chimeric RNAs by the dual luciferase assay system. The co-overexpression of *miR-23b* and *miR-27b* significantly reduced the luciferase activity of Renilla_*FOPX2*-3′UTR (Figure 7B). Two mutant vectors (MT1 and MT2), which harbored the six-point mutations within each binding sites for *miR-23b* or *miR-27b* (Figure 7A), slightly, but significantly, rescued each microRNA-reduced luciferase activity, suggesting that FOXP2 may be regulated, at least in part, by *miR-23b* and/or *miR-27b* (Figure 7C,D).

### 2.8. Promotion of Cell Migration in the FOXP2 Knockdown Cells

**Figure 8:**
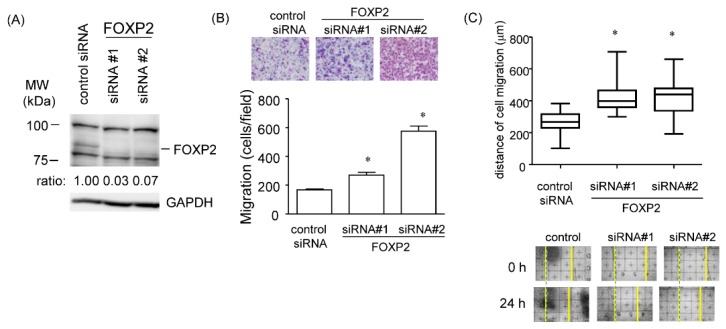
Promotion of cell migration in FOXP2 knockdown cells. (**A**) Amounts of FOXP2 protein in non-MG cells treated with two siRNAs targeted to distinct FOXP2 sequences were measured using western blotting with GAPDH as a loading control. (**B**) Upper panels show representative images of Diff-Quick staining in four experiments of transwell migration assays, and a lower graph shows quantification of cell migration expressed by cell counting. Data are expressed as the mean ± standard deviation (SD; n = 4). * Statistically significant difference versus control (unpaired Student’s *t*-test, *p* < 0.05). (**C**) Scratch assay was performed in non-MG cells with FOXP2-siRNA treatment. Migration distance was measured 24 h after cells were scratched. Representative images were shown in lower panels. Data are expressed as the mean ± standard deviation (SD; n = 4). * Statistically significant difference versus control (unpaired Student’s *t*-test, *p* < 0.05). MW, molecular weight.

FOXP2 was initially identified as the genetic factor of speech disorder, and its mutations lead to speech and language disorder. Recently, several lines of evidence have revealed that the down-regulation of FOXP2 may be associated with tumor initiation, development, or metastasis [21,22,23,24]. To investigate the effects of FOXP2 on cell migration, we employed transwell and scratch assays. The non-MG cells were transfected with two different siRNAs (#1 and #2) targeting FOXP2 for 36 h. The two independent siRNAs efficiently reduced FOXP2 protein levels (Figure 8A). The transwell assay showed that inhibition of FOXP2 expression increased the number of migrated cells, and the scratch assay also showed that FOXP2 siRNA treatment significantly facilitated cell migration (Figure 8B,C). These data suggested that FOXP2 may play the role of a migration inhibitory factor in the non-MG cells.

## 3. Discussion

Extensive genome-wide analyses have elucidated that genetic heterogeneity is observed as a common feature of various tumors. During cancer development, genetic heterogeneity contributes to cancer adaptation to their surrounding microenvironments. Cell migration is an essential step in cancer metastasis. In the present study, we found up-regulation of *miR-23b/27b/24* cluster expression in a subpopulation with high migration capacity using the transwell-based migrated cell selection. Further analyses showed that the overexpression of either or both *miR-23b* and *miR-27b* may promote cell migration via potentially targeting FOXP2.

Intratumor heterogeneity is a fundamental mechanism to cope with diverse surrounding microenvironments, such as immune cells and stroma cells. Sato et al. reported that even a cell line is composed of highly heterogeneous cells [25]. They showed that certain subpopulation of cultured cells with immortal phenotype could be putative cancer stem cells. We successfully isolated a subpopulation with high migration capacity by transwell-based migrated cell selection. We were interested in molecules, which introduce distinct subpopulations. In this study, we focused on miRNA regulation involved in acquisition of cell migration capacity. Recently, as numerous studies revealed, the altered expression of miRNAs is involved in cancer development and prognosis [26]. We showed here that *miR-23b/27b/24* cluster may be involved in the acquisition of migratory phenotype. The *miR-23b/27b/24* cluster belongs to the hetero-seed clusters, which have distinct seed sequences [27]. Although several reports have revealed the functions of individual miRNAs belonging to *miR-23b/27b/24* cluster, there are few and controversial evidences which mentioned the functions of *miR-23b/27b/24* cluster. The expression of the *miR-23b/27b/24* cluster is significantly reduced in prostate cancer tissues, and the overexpression of *miR-27b* and *miR-24* inhibits cell invasion and migration in prostate cancer cell lines [28]. In contrast, in breast cancer, the *miR-23b/27b/24* functions in tumor progression, and promotes metastasis [12,29]. However, the function of *miR-23b/27b/24* cluster in colon cancer cells has not been studied. The present study is the first report about the function of *miR-23b/27b/24* cluster in colon cancer cells migration. Interestingly, the overexpression of either or both *miR-23b* and *miR-27b* promoted cell migration, but *miR-24* had an opposite effect in cell migration, suggesting that the expression balance of individual miRNAs, belonging to *miR-23b/27b/24* cluster, may be associated with distinct phenotypes.

Approximately 40% of mammalian miRNAs are located within introns of host genes, and their expression is transcriptionally linked to their host gene expression and processed from the same primary transcript. In the human genome, most intronic miRNAs show correlated expression with their host genes, and intronic miRNAs often support the function of their host genes by synergistic mediating and antagonistic regulatory effects [30]. We found increased levels of *miR-23b*, *miR-27b*, and *miR-24* expression consistent with the mRNA levels of *C9orf3* (a host gene in the *miR-23b/27b/24* cluster), which encodes aminopeptidase O (AOPEP), a metallopeptidase [31]. However, the function of AOPEP in cancer development has not been elucidated. It is of interest to uncover the functional relationship between AOPEP and the *miR-23b/27b/24* cluster in future investigations. We found that the expression of *C9orf3* mRNA harboring exons 10 to 15 was upregulated in MG cells, suggesting that a novel transcriptional start site may exist upstream of exon 10. Strikingly, we demonstrated that E2F1 biding sites are essential sequences for the promoter activity of a short *C9orf3* transcript using promoter assays and ChIP. E2F1 is a cell cycle-specific transcription factor that binds to promoters of diverse downstream genes. Although E2F1 has pleiotropic roles in tumorigenesis [32,33], several lines of evidence have revealed that E2F1 promotes cell migration and aggressiveness in prostate and colorectal cancer cells [18,20]. *C9orf3* may be a novel downstream target of E2F1 associated with cell migration and cancer development.

The clustered miRNAs are often conserved evolutionarily and are likely to cooperatively regulate functionally related target genes [27]. For example, BCL2L11 is targeted by multiple miRNAs of the *miR-17/92* cluster [34,35]. Because either or both *miR-23b* and *miR-27b* promote cell migration, we focused on those target genes that are cooperatively regulated by both *miR-23b* and *miR-27b*. Based on miRDB, we found FOXP2 as a common target gene of *miR-23b* and *miR-27b*. FOXP2 is a member of the Forkhead family of transcription repressor that regulates speech and learning in humans. Luciferase assay showed that mutation of *miR-23b* or *miR-27b* binding site could not fully recover luciferase activity; suggesting that other factors, regulated by *miR-23b* and *miR-27b*, may be involved in FOXP2 regulation. The function of FOXP2 was originally investigated in neuronal network development including migration or morphological neuronal cell changes. Recently, FOXP2 was also shown to be involved in cancer development. FOXP2 negatively regulates stem cell-associated factors—such as c-MYC, OCT-4, and CD44—in breast cancer, resulting in a putative tumor/metastasis suppressor [23]. In addition, the expression of FOXP2 is mediated by miR-23a, a member of the *miR-23/27/24* family [21]. Furthermore, Abu-Remaileh et al. reported that *FOXP2* mRNA levels were significantly decreased in colon cancer samples [36]. We demonstrated that FOXP2 was downregulated in mRNA and protein levels in the MG cells and FOXP2 knockdown enhanced cell migration. These results are consistent with those of previous reports that FOXP2 promotes cell migration. To the best of our knowledge, direct targets of FOXP2 have not been investigated and further studies are needed to elucidate the function of FOXP2 in cancer development or metastases.

In this study, we established the cell selection procedure regarding cell migration. It remains unclear as to whether other processes of EMT or metastasis—such as adhesion, invasion, and attachment—are promoted in the MG cells. Efforts to elucidate the functions of miR-23b/27b/FOXP2 in vivo are currently underway.

## 4. Materials and Methods

### 4.1. Cell Culture and Isolation of a Subpopulation with High Cell Migration Capacity

A subpopulation with high migratory capacity, MG cells, were isolated by a transwell-based selection method [13]. In brief, for the isolation of a subpopulation with cell migration capacity, we established the following selection procedures using the transwell migration assay (the modified Boyden Chamber assay [37]). HCT116 cells were seeded in serum-free media on the upper side of a transwell chamber (Becton Dickinson, Franklin Lakes, NJ, USA) and allowed to migrate towards the media containing 10% of fetal bovine serum (FBS). Both cells remaining on the upper membrane (non-MG cells) and cells migrating to the lower side of the membrane (MG cells) were respectively collected. To concentrate the subpopulations, this selection procedure was employed five more times. After this selection procedure, two subpopulations were isolated; one was the non-MG cells remaining on the upper membrane, and another was the MG cells preferentially migrating to the lower membrane. These cells were cultured in DMEM (Nacalai Tesque, Kyoto, Japan) supplemented with 10% (vol/vol) heat-inactivated FBS at 37 °C in 5% CO_2_.

### 4.2. Cell Counting, Cell Migration Assay and Scratch Assay

Cells were seeded in tissue culture plates, and the numbers of growing cells were counted using a hematocytometer. Migration of colon cancer cells were examined using 8-μm pore size polycarbonate transwell filters (Becton Dickinson). After 48 h serum starvation, cells were seeded in serum-free media on the upper side of a transwell chamber and allowed to migrate towards the media containing 10% FBS for 48 h. After the incubation period, cells on the lower side of the membrane were fixed, stained with Diff-Quick stain (Sysmex, Kobe, Japan) and counted. The migration indices were calculated as the mean number of cells in 5 random fields at 20× magnification. For the scratch assay (wound healing assay), cells were seeded onto 24-well tissue culture plates and maintained at 37 °C and 5% CO_2_ for 24 h, and then, plasmids or mimic miRNAs were transiently transfected into the cells. To permit the formation of a confluent monolayer, the cells were incubated for 36 h after transfection. These confluent monolayers were then scratched with a cell scratcher (Asahi Techno Glass Co., Ltd., Tokyo, Japan). Culture medium was then immediately replaced with a fresh culture medium (10% FBS) to remove any dislodged cells. Cell migration ability was determined by measuring the migration distance in 5 points per sample at 24 h after the scratch. All scratch assays were performed in quadruplicate.

### 4.3. Small Interference RNAs (siRNAs)

We used siRNAs (Hs_FOXP2_12 and 13 HP validated siRNAs; Qiagen, Chatsworth, CA, USA) to knock down *FOXP2* mRNA. A negative control siRNA (AllStars Negative Control siRNA) was obtained from Qiagen.

### 4.4. Overexpression of miRNA Mimics and Inhibitors

miRCURY LNA miRNA mimics (has-miR-23b, Product No. 472291-001; has-miR-27b, Product No. 470553-001) and mimic negative control were obtained from Exiqon (Vedbaek, Denmark). mirVana miRNA mimic (has-miR-24-3p; MC10737) was obtained from Ambion (Austin, TX, USA). miRCURY LNA miRNA inhibitors (has-miR-23b, Product No. 4102299; has-miR-27b, Product No. 4103307; has-miR-24-3p, Product No. 4101706; negative control A) were obtained from Exiqon. HCT116, DLD1, RKO, and HT-29 cells were transfected with miRNA mimics or inhibitors at 10 nM using Lipofectamine RNAiMAX (Thermo Fisher Scientific, Waltham, MA, USA).

### 4.5. Quantitative Real-Time Reverse Transcription-PCR (qPCR)

Total RNAs, including miRNAs, were extracted from cells using RNA iso plus reagent (Takara, Otsu, Japan). For analysis of mRNA expression levels, 1 μg of isolated RNA was reverse-transcribed using ReveTra Ace qPCR RT Master Mix (Toyobo, Osaka, Japan). *C9orf3*, *CLCN3*, *KCNK2*, *FOXP2, PKIA*, *SEC24A*, *TRIL*, and *ZDHHC17* mRNA levels were measured using SYBR Green Master Mix and Applied Biosystems 7500 Real-time System (Applied Biosystems, Foster City, CA, USA). The sequences of primer sets are provided in Table S1. mRNA levels were measured by the comparative ΔΔCt method using g*lyceraldehyde-3-phosphate dehydrogenase* (*GAPDH*) mRNA as a control and expressed as values relative to the indicated control sample. For analysis of miRNA expression levels, isolated RNAs were cleaned using miRNeasy mini kit (Qiagen, Hilden, Germany) and reverse-transcribed using miRNURYLNA Universal RT kit according to the manufacturer’s protocol. Locked nucleic acid (LNA) PCR primer sets (Exiqon) targeting hsa-miR-23b-3p (Product No. 204790), hsa-miR-27b-3p (Product No. 205915), hsa-miR-24-3p (Product No. 204260), and hsa-miR-10a-5p (Product No. 204788) were used to detect miRNA expression. miRNA levels were measured by the comparative ΔΔCt method using RNU48 as a control and expressed as values relative to the indicated control sample.

### 4.6. Analysis of Global miRNA Expression

Total RNAs were extracted from cells using miRNeasy kit (Qiagen) according to the manufacturer’s protocol. RNA concentration and purity were determined by NanoDrop ND-1000 spectrophotometer (NanoDrop Technologies, Wilmington, DE, USA). Purified RNA quality was assessed by Agilent 2100 Bioanalyzer using an RNA 6000 Nano Labchip kit (Agilent Technologies, Santa Clara, CA, USA), and RNA samples with above 8.5 RNA integrity number (RIN) were used for further study. RNA samples were used to measure miRNA expression profiles using a human miRNA microarray (G4470C; Agilent), containing 939 miRNA probes, as previously described. These data were analyzed using GeneSpring 11.5.1 (Agilent). We selected miRNAs with fluorescence intensities 50 in at least half of the RNA samples, resulting in the detection of 56 miRNAs in the samples. All primary and uniformly processed sequence data generated in this study are available at the NCBI Gene Expression Omnibus (GEO; http://www.ncbi.nlm.nih.gov/geo/) under accession number GSE141240.

### 4.7. miRNA Luciferase Reporter Assay

The fragment of the 3′UTR of *FOXP2* was amplified from the cDNA of HCT116 cells using primer sets listed in Table S1. The fragment of *FOXP2* 3′UTR was subcloned into psiCHECK-2 vector (Promega, Madison, WA, USA) using XhoI and NotI restriction sites. HCT116 cells were cultured on 24-well plates, and then psiCHECK-2 constructs with various site-directed mutations were co-transfected with either *miR-23b* or *miR-27b*, or both. Twenty-four hours after the transfection, cells were harvested and the firefly and Renilla luciferase activities were measured using the Dual-Luciferase Reporter Assay System (Promega).

### 4.8. Western Blotting

Whole-cell lysates were prepared in a RIPA buffer (10 mM Tris-HCl, pH 7.4; 1% Nonidet P-40; 1 mM EDTA; 0.1% SDS; 150 mM NaCl) containing a protease inhibitor (Nacalai Tesque) and phosphatase inhibitor cocktail (Sigma). Rabbit polyclonal anti-E2F1 (1:1000; Cell Signaling Technology, Danvers, MA, USA), rabbit polyclonal anti-FOXP2 (1:1000; #3742, Cell Signaling Technology), or mouse monoclonal anti-GAPDH (1:5000; Santa Cruz Biotechnology) antibody was used. Full-length western blots were shown in Figures S6–S8.

### 4.9. Promoter Activity Assay

The 5′-flank of the human *C9orf3* gene was cloned into the pGL4.21-basic luciferase reporter vector (Promega). In brief, the first PCR was performed using human genomic DNA as a template. The *C9orf3* proximal promoter region was amplified using primer sets listed in Table S1. The amplified products were subcloned into the pGL4.21-basic vector using KpnI and HindIII restriction sites. HCT116 cells (1.0 × 10^5^) were cultured on 24-well plates, and then pGL-4.21 luciferase constructs with various site-directed mutation or deletion (100 ng) were cotransfected with pGL4.74 vector (50 ng) using X-tremeGENE HP DNA transfection reagent (Roche, Basel, Switzerland). Twenty-four hours after the transfection, cells were harvested, and the firefly and Renilla luciferase activities were measured using the Dual-Luciferase Reporter Assay System (Promega).

### 4.10. Chromatin Immunoprecipitation (ChIP) Assay

ChIP assays were performed using the Chromatin Immunoprecipitation Assay Kit (Millipore, Burlington, CA, USA). Briefly, HCT116 cells were fixed with 1% formaldehyde in phosphate-buffered saline (PBS) for 10 min and then washed twice with ice-cold PBS. These cells were resuspended in SDS lysis buffer, incubated for 10 min on ice, and then sonicated. Immunoprecipitation was carried out overnight at 4 °C using 3 μg antibody against E2F1 (Cell Signaling Technology). Normal rabbit IgG was used to assess the non-specific reactions. Immune complexes were collected with protein A agarose/salmon sperm DNA. Cross linking between proteins and DNA was reversed according to the manufacturer’s protocol. Protein-bound DNA was extracted with phenol/chloroform/isoamyl alcohol. The extracted DNA was amplified by PCR (35 cycles; denaturing at 98 °C for 10 s, annealing at 55 °C for 30 s, and extension at 72 °C for 1 min) using the following primers: for the *C9orf3* primer set 1 sequence between −209 and +84 bp, 5′-GCGGCCGCTATACCTTTA-3′ and 5′-GCGCGACGCTCCCTCTGCAG-3′; for the *C9orf3* primer set2 sequence between −1241 and −1110 bp, 5′-TCCCTCATTTGAGCAGACTTGT-3′ and 5′-ACACAGAGGAGCTTAATGGAGAC-3′. The nuclear chromatin DNA from HCT116 cells (input) was used as a positive control for PCR.

### 4.11. Data Analysis

RNA-seq and clinical data of colorectal adenocarcinoma (CORDREAD) from The Cancer Genome Atlas (TCGA) were analyzed using the UCSC Xena web interface [38].

## 5. Conclusions

In conclusion, the present study demonstrated a novel role of the *miR-23b/27b/24* cluster in cell migration through targeting FOXP2, using the transwell-based separated cells. These data suggest that a novel miR-23b/27b/FOXP2 link may play, at least in part, an important role in the migratory activity in cancer development and might be a potential target for the inhibition of cancer metastases.

## Figures and Tables

**Table 1:** MicroRNAs (miRNAs) with >1.5-fold significant expression change in the migrated cells (MG cells).

miRNA	Change Relative to Upper Cells ^(1)^ (Fold-Change)	mirBase Accession No.
*hsa-miR-10a*	2.119	MIMAT0000415
*hsa-miR-23b*	2.610	MIMAT0000418
*hsa-miR-27b*	1.837	MIMAT0000419
*has-miR-1274b* ^(2)^	1.511	MIMAT0005938

^(1)^ Values are expressed as fold changes compared with the values in the non-MG cells. ^(2)^ These miRNAs were removed as dead entries on the miRBase (Release 21).

## Supplementary Materials

The following are available online at https://www.mdpi.com/2072-6694/12/1/174/s1. Table S1: Primer sets used for qPCR and 5′-RACE; Figure S1: mRNA expression of surface markers of colorectal cancer stem cells; Figure S2: Identification of transcriptional start sites by 5′-rapid amplification of cDNA end assay; Figure S3: Effects of DNA methylation on *C9orf3* and *miR-23b/27b/24* expression; Figure S4: Effects of knockdown of E2F1 in non-MG cells on miR-23b/27/24 expression; Figure S5: Effects of miR-23b/27b/24 on cell migration; Figures S6–S8: Full-length western blots.
