# Supplementary material for: The MicroRNA-23b/27b/24 Cluster Facilitates Colon Cancer Cell Migration by Targeting FOXP2
Source: Cancers (Basel). 2020 Jan 10;12(1):174. doi: 10.3390/cancers12010174 (PMC7017312; doi:10.3390/cancers12010174)
Supplement: Supplementary file 1 [file cancers-12-00174-s001.pdf]

Supplementary material

The MicroRNA-23b/27b/24 Cluster Facilitates Colon Cancer Cell Migration by Targeting FOXP2

Kensei Nishida, Yuki Kuwano, Kazuhito Rokutan

Materials and Methods

Rapid amplification of cDNA ends (RACE) analyses

Total RNA was isolated from MG cells and subjected to 5′-RACE analysis using a SMARTer RACE 5′/3′ Kit (Clontech Laboratories) according to the manufacturer's protocol. Reverse transcription was performed using Powerscript reverse transcriptase with the 5′-RACE cDNA synthesis primer. Details regarding the gene-specific reverse primers for 5′-RACE are listed in Table S1. The polymerase chain reaction (PCR) products were gel purified and cloned into the pRACE vector (Clontech Laboratories), and transformed into E. coli DH5a cells (Toyobo, Osaka, Japan) for Sanger sequencing.

DNA demethylation

Cells were cultured in medium with 10% FBS containing concentrations of 5 μM 5-azacytidine (Nacalai Tesque) for 48 h. After treatment with 5-azacytidine, total RNA was extracted from the cells. For analysis of miRNA expression levels, isolated RNAs were cleaned up using miRNeasy mini kit (Qiagen, Hilden, Germany) and reverse-transcribed using miRNURYLNA Universal RT kit according to the manufacturer's protocol. LNA PCR primer sets (Exiqon) targeting hsa-miR-23b-3p (Product No.204790), hsa-miR-27b-3p (Product No.205915), hsa-miR-24-3p (Product No.204260) were used to detect miRNA expression. miRNA levels were measured by the comparative ΔΔCt method using RNU48 as a control and expressed as values relative to the indicated control sample.

Supplementary Table S1: Primer sets used for qPCR and primers for 5’-RACE

Primers for qPCR

Targets		primer sequences (5' - 3')
C9orf3 Ex1-2	forward	CGGCTGAGACAGGAGACTG
	reverse	GAGAGGCAGGTCATCTCTGG
C9orf3 Ex3-4	forward	ACGCAGCTTTGGGAAGAGT
	reverse	TTCTGTCCAGCATCCCACT
C9orf3 Ex10-12	forward	GCCGAGGTCACGAAATGGAT
	reverse	TCCAGAAGCAAGACCAGCTG
C9orf3 Ex13-15	forward	CCATCGGTGGTGTGAACTCA
	reverse	GAATCTTGCTGTGGTCTTTCCT
C9orf3 Ex14-15	forward	CGGACCAAGGAGCAGATG
	reverse	GAATCTTGCTGTGGTCTTTCCT
E2F1	forward	TCTATGACATCACCAACGTCCT
	reverse	CTGGGTCAACCCCTCAAG
CLCN3	forward	GGTGCTATCAGCTGCCTCA
	reverse	ATAGCTAACCTCTTCCAGGCTAAA
KCNK2	forward	TGGAGTTGTGACTTGGCTGT
	reverse	AATCTGCATTTTTCCATGTGC
FOXP2	forward	GCAGCTCTTAATGCCAGTTTG
	reverse	AGGCCACTGGATGCATTATT
PKIA	forward	AGTGGACCCCATCCCTAAAC
	reverse	TTTCTGTTTCTCTCCCAGATCAA
SEC24A	forward	CATGCCTCGGGCTTAATG
	reverse	AGGGAGGAGGCTAACGACTT
TRIL	forward	AGGGACTCCACTGGTTTGGT
	reverse	GTTAAACTTTACCACCTGTTTACATCC
ZDHHC17	forward	AATGCAGATTACAGGTATTAAAGCAA
	reverse	TGGTACTTGGAAATACTGAACATCA
ZBTB34	forward	CTGCCCCTCAGTGACAAGTAT
	reverse	ATAGTCAGGGCAGCAACCA
ALDH1A1	forward	TGTTAGCTGATGCCGACT
	reverse	TTCTTAGCCCGCTCAACACT
EPCAM	forward	GCTGGTGTGTGAACACTGCT
	reverse	ACGCGTTGTGATCTCCTTCT
CD133	forward	CTGGGGCTGCTGTTTATTATTCTG
	reverse	ACGCCTTGTCCTTGGTAGTGTTG
CD24	forward	ACCCACGCAGATTTATTCCA
	reverse	ACCACGAAGAGACTGGCTGT

CD26	forward	CGTTACATGGGTCTCCCAAC
	reverse	CAGGGCTTTGGAGATCTGAG
POU5F1	forward	GAAGGATGTGGTCCGAGTGT
	reverse	GTGAAGTGAGGGCTCCCATA
SOX2	forward	AACCCCAAGATGCACAACTC
	reverse	CGGGGCCGGTATTTATAATC
SOX9	forward	AGTACCCGCACTTGCACAAC
	reverse	CGTTCTTCACCGACTTCCTC
GAPDH	forward	AGCCACATCGCTCAGACAC
	reverse	GCCCAATACGACCAAATCC

Gene-specific primers for 5’ RACE

Name		primer sequences (5' - 3')
GSP_RT	reverse	GGCGTCCTCACTCAC
GSP1	reverse	CCACCTGGGCTGAGGACCTA
GSP2	reverse	CTGCTGTCTGGCGTCCTCAC

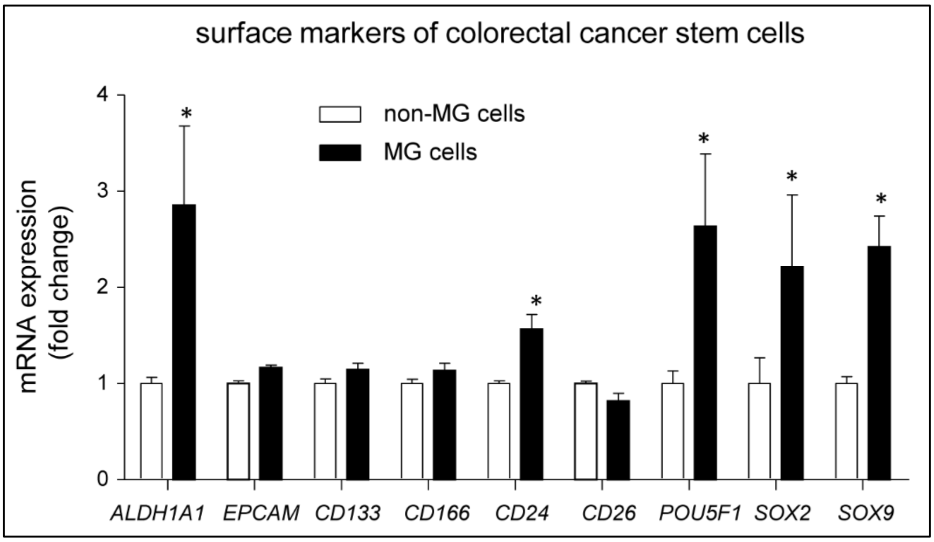

Figure S1. mRNA expression of surface markers of colorectal cancer stem cells. mRNA levels of *ALDH1A1*, *EPCAM*, *CD133*, *CD24*, *CD26*, *POU5F1*, *SOX2*, and *SOX9* were determined using RT-qPCR in non-MG cells and MG cells. *GAPDH* was used as an endogenous control. Data are expressed as the mean fold changes ± standard deviation (SD; n = 4), compared with those in the non-MG cells. *Statistically significant difference versus non-MG cells (unpaired Student’s t-test, P <0.01).

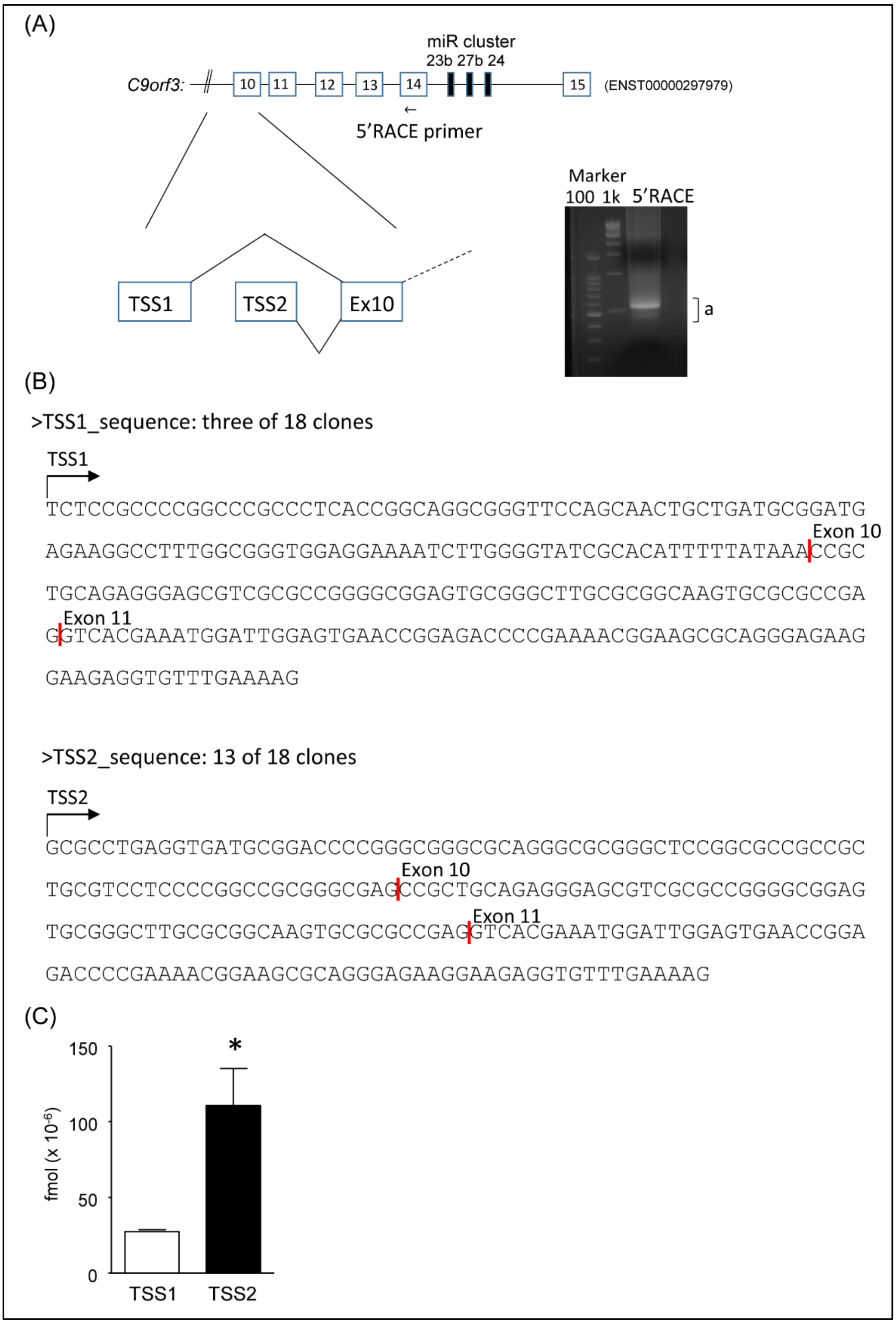

Figure S2. Identification of transcriptional start sites by 5′-rapid amplification of cDNA end assay. (**A**) Scheme diagram of the gene-specific primers used for 5′-RACE experiment. Two transcript start site (TSS) were detected by 5′RACE assay. Electrophoretic analysis of PCR amplification products. (**B**) Nucleotide sequences of the PCR products. Red lines indicate the junctions between different exons. (**C**) Absolute RT-qPCR were performed using TSS1 and TSS2 specific primers. Data are expressed as the mean ± standard deviation (SD; n = 4). *Statistically significant difference between TSS1 and TSS2 expression levels (unpaired Student’s t-test, P <0.05).

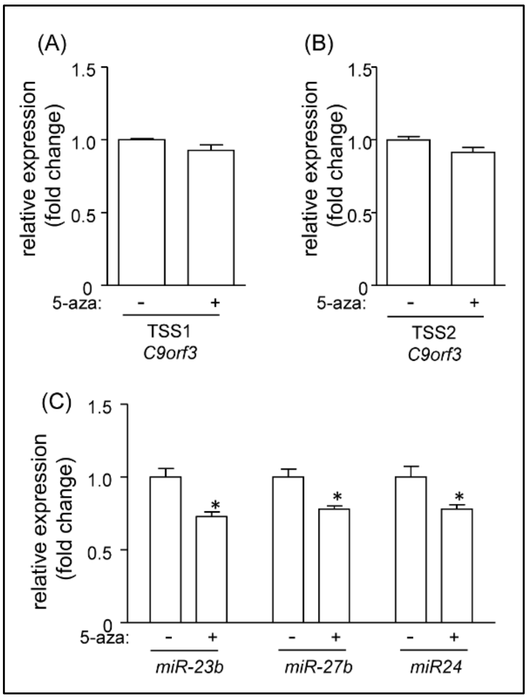

Figure S3. Effects of DNA methylation on C9orf3 and miR-23b/27b/24 expression. (**A** to **C**) Relative expression levels of TSS1 and TSS2, and miR-23b, miR-23b/27b/24 miRNAs in non-MG cells with 5-azacytidine (5-aza) treatment were measured by RT-qPCR. GAPDH and RNU48 were used as an endogenous control. Data are expressed as the mean fold changes ± standard deviation (SD; n = 4), compared with non-treated non-MG cells. *Statistically significant difference versus non-treated non-MG cells (unpaired Student's t-test, P <0.05).

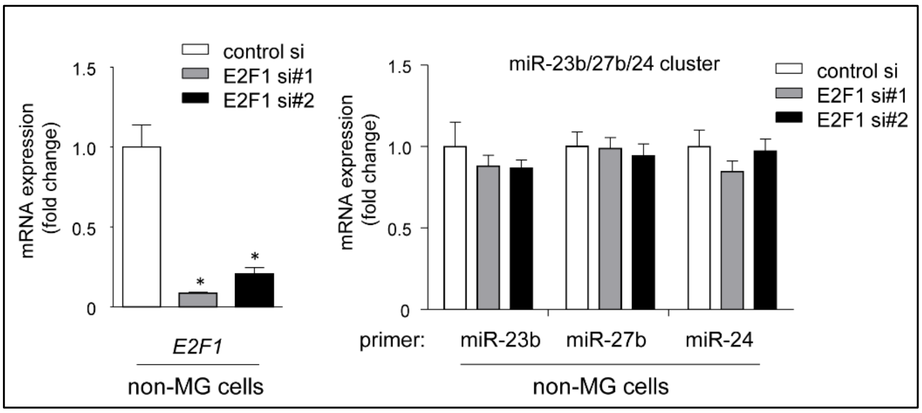

Figure S4. Effects of knockdown of E2F1 in non-MG cells on miR-23b/27/24 expression. **(A)** Two siRNAs targeted for distinct E2F1 sequences were transfected to the non-MG cells. mRNA levels of E2F1 in E2F1 siRNAs-treated cells were measured by RT-qPCR using *GAPDH* as an endogenous control. (**B**) Relative expression levels of *C9orf3* and *miR-23b/27b/24* miRNAs in non-MG cells after treatment with E2F1-siRNAs were measured by RT-qPCR. *RNU48* were used as an endogenous control. Data are expressed as the mean ± standard deviation (SD; n = 4). *Statistically significant difference versus control cells (unpaired Student's t-test, P <0.05)

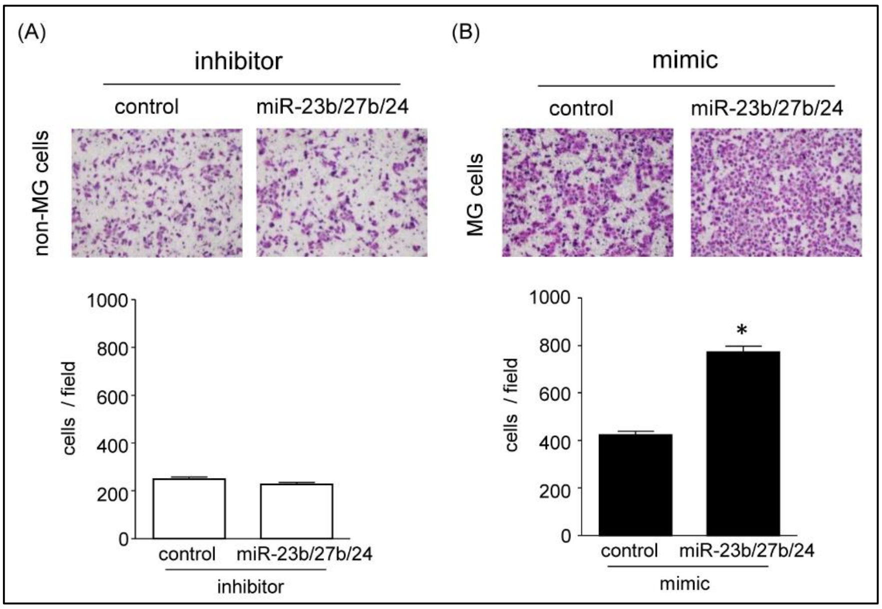

Figure S5. Effects of miR-23b/27b/24 on cell migration. (**A** and **B**) Transwell migration (n = 4) assays were performed in mixed mimic or inhibitor of microRNAs (*miR-23b*, *miR-27b* and *miR-24*)-transfected HCT116 cells. Upper panels show representative images of Diff-Quick staining in four experiments of transwell migration assays, and a lower graph shows quantification of cell migration expressed by cell counting. *Statistically significant difference versus control cells (unpaired Student's t-test, P <0.05)

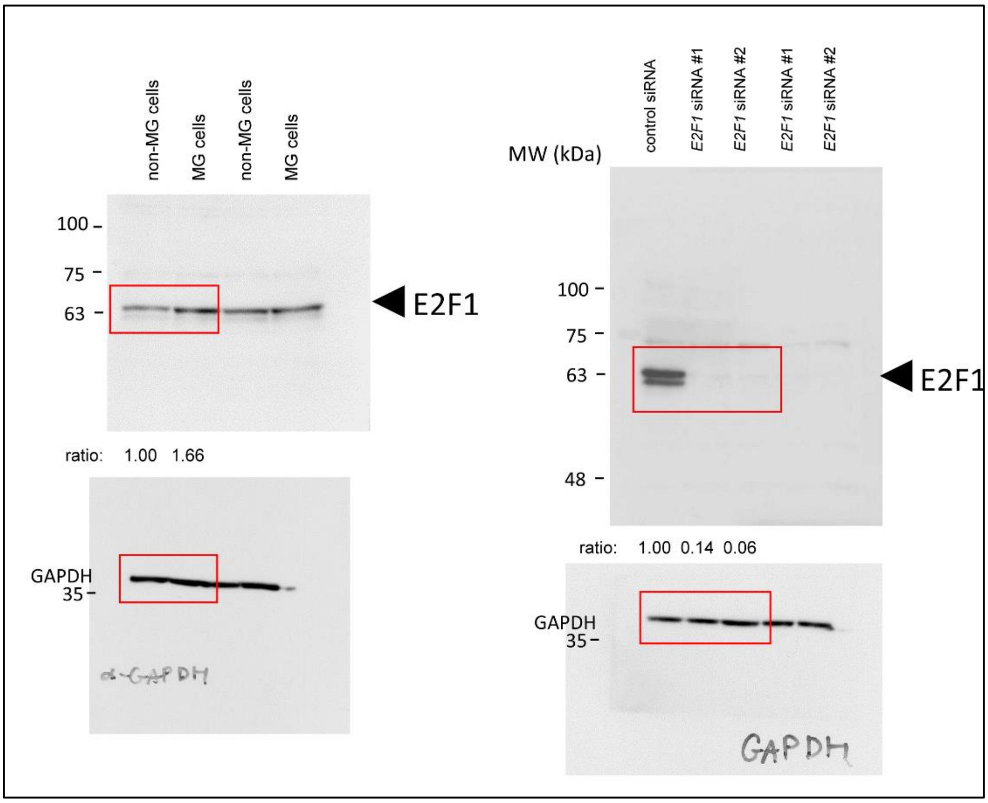

Figure S6. Full-length western blots for Figure 2D and 3A. The results of western blotting with an anti-E2F1 antibody were quantified by densitometry, and the ratio of E2F1 to GAPDH is shown.

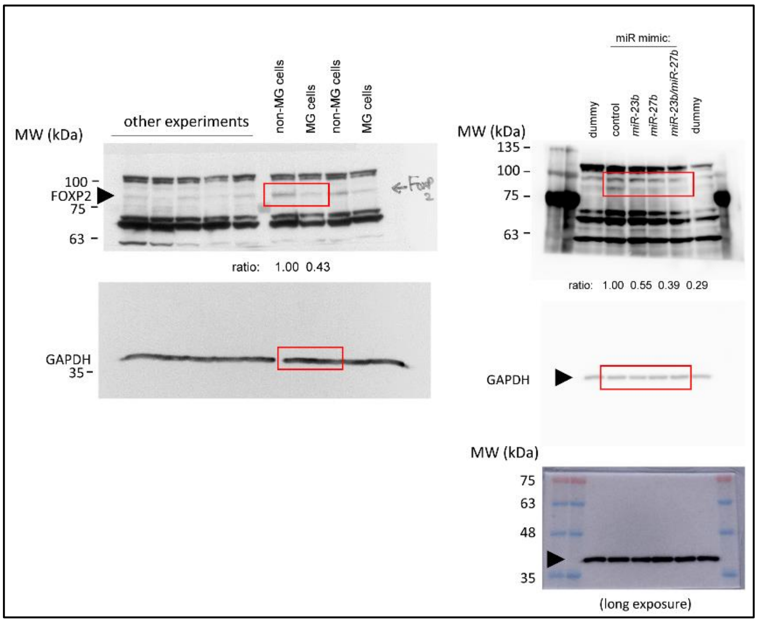

Figure S7. Full-length western blots for Figure 6C and 6E. The results of western blotting with an anti-FOXP2 antibody were quantified by densitometry, and the ratio of FOXP2 to GAPDH is shown.

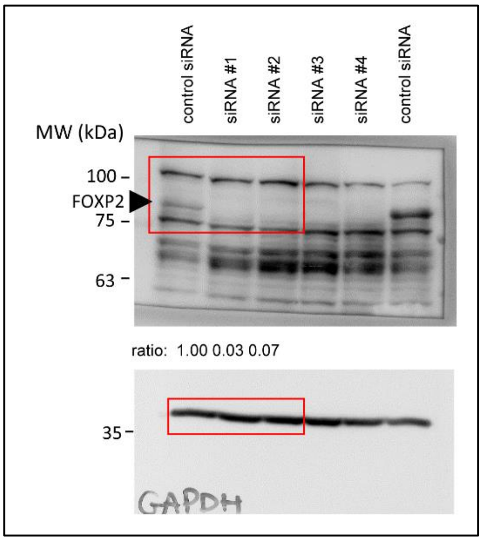

Figure S8. Full-length western blots for Figure 8A. The results of western blotting with an anti-FOXP2 antibody were quantified by densitometry, and the ratio of FOXP2 to GAPDH is shown.

© 2019 by the authors. Licensee MDPI, Basel, Switzerland. This article is an open access article distributed under the terms and conditions of the Creative Commons Attribution (CC BY) license (http://creativecommons.org/licenses/by/4.0/).
